# Establishment of a Public Mental Health Database for Research Purposes in the Ferrara Province: Development and Preliminary Evaluation Study

**DOI:** 10.2196/45523

**Published:** 2023-08-09

**Authors:** Maria Ferrara, Elisabetta Gentili, Martino Belvederi Murri, Riccardo Zese, Marco Alberti, Giorgia Franchini, Ilaria Domenicano, Federica Folesani, Cristina Sorio, Lorenzo Benini, Paola Carozza, Julian Little, Luigi Grassi

**Affiliations:** 1 Institute of Psychiatry, Department of Neuroscience and Rehabilitation, University of Ferrara, Ferrara, Italy; 2 Integrated Department of Mental Health and Pathological Addictions, Ferrara Local Health Trust, Ferrara, Italy; 3 Department of Psychiatry, Yale School of Medicine, New Haven, CT, United States; 4 Department of Engineering, University of Ferrara, Ferrara, Italy; 5 Department of Chemical, Pharmaceutical and Agricultural Sciences, University of Ferrara, Ferrara, Italy; 6 Department of Mathematics and Computer Science, University of Ferrara, Ferrara, Italy; 7 Department of Physics, Informatics and Mathematics, University of Modena and Reggio Emilia, Modena, Italy; 8 School of Epidemiology and Public Health, University of Ottawa, Ottawa, ON, Canada

**Keywords:** mental health, psychosis, epidemiology, electronic health registry, health care, machine learning, medical health records, electronic health records, clinical database, support, mental disorder, social determinants, mental health care, resource utilization

## Abstract

**Background:**

The immediate use of data exported from electronic health records (EHRs) for research is often limited by the necessity to transform data elements into an actual data set.

**Objective:**

This paper describes the methodology for establishing a data set that originated from an EHR registry that included clinical, health service, and sociodemographic information.

**Methods:**

The Extract, Transform, Load process was applied to raw data collected at the Integrated Department of Mental Health and Pathological Addictions in Ferrara, Italy, from 1925 to February 18, 2021, to build the new, anonymized Ferrara-Psychiatry (FEPSY) database. Information collected before the first EHR was implemented (ie, in 1991) was excluded. An unsupervised cluster analysis was performed to identify patient subgroups to support the proof of concept.

**Results:**

The FEPSY database included 3,861,432 records on 46,222 patients. Since 1991, each year, a median of 1404 (IQR 1117.5-1757.7) patients had newly accessed care, and a median of 7300 (IQR 6109.5-9397.5) patients were actively receiving care. Among 38,022 patients with a mental disorder, 2 clusters were identified; the first predominantly included male patients who were aged 25 to 34 years at first presentation and were living with their parents, and the second predominantly included female patients who were aged 35 to 44 years and were living with their own families.

**Conclusions:**

The process for building the FEPSY database proved to be robust and replicable with similar health care data, even when they were not originally conceived for research purposes. The FEPSY database will enable future in-depth analyses regarding the epidemiology and social determinants of mental disorders, access to mental health care, and resource utilization.

## Introduction

Electronic health records (EHRs) assemble and enable access to large volumes of clinical and sociodemographic data that are routinely collected by local health authorities. EHRs offer a unique opportunity to conduct research on various topics, including, among others, the patterns of health care resource use and factors that influence the course and outcomes of mental disorders in large, representative samples [1,2]. EHRs can be linked to data related to census and geolocalization information [3]; such investigations span the epidemiology of mental disorders, hospitalization rates, morbidity, and mortality.

The breadth and nature of information represented in the sample of EHRs in the mental health sector make such information particularly suitable for using artificial intelligence (AI) and machine learning techniques, in addition to traditional methods (eg, linear regression models), in order to increase the potential for research on social and clinical factors [4].

Applications that use AI take advantage of AI's ability to process large amounts of data in order to extract information or identify underlying patterns of relationships that conventional methods may overlook [5]. AI may be particularly suitable for the investigation of large amounts of clinical data, thanks to (1) the flexibility and scalability of AI techniques, which are higher than those of traditional methods, and (2) the ability of AI to consider all of the available predictors (ie, not only a subset), which makes AI and, in particular, machine learning suitable for performing tasks such as classification, prediction, and resource optimization [5,6].

Indeed, in recent years, the use of AI techniques in mental health care research have rapidly increased, including its use to identify a disease at its earliest stages, predict illness onset in vulnerable individuals, study illness progression, optimize treatment, and discover novel therapeutic agents [7,8].

As of yet, there are few examples (mainly from the United States) of how data collected from EHRs can be successfully adapted for analysis with AI. For example, Hughes et al [9] analyzed clinical variables in the EHRs of 81,630 adults from 2 academic medical centers in Boston, Massachusetts (spanning 10 years) and identified predictors of treatment response for major depressive disorders.

Xu et al [10] compiled a data set of 11,275 patients from 5 large medical centers across New York City by using EHR data collected between 2008 and 2017; they used machine learning methods to identify markers of depression phenotypes to inform clinical decisions about patients’ care. Pradier et al [11] analyzed a data set of 67,807 individuals to predict the risk of receiving a misdiagnosis of bipolar disorder among individuals with depression, using only information retrieved from EHRs. Perlis et al [12] applied natural language processing to classify the mood states of 127,504 patients, using data from an EHR.

In order to fully exploit the potential of EHRs for mental health research however, important issues need to be considered. One preliminary, controversial issue is whether the use of EHR data should be restricted to the purpose for which they were collected [13]. Indeed, privacy constraints, data security, and overall ethics regulation must be taken into account when considering whether to use EHRs for research purposes [6]. Nonetheless, nowadays, medical data that were originally collected for purposes other than research are being used to study health phenomena in many different fields, including mental health, substance use, noncommunicable diseases (eg, cancer), and health behaviors (eg, cancer screening) [14]. A further challenge is that data may not be homogeneous or may not be collected systematically, and most data are not derived from structured scales or questionnaires. The adaptation of the EHR represents the first necessary step to planning research projects that include models for predicting health resource utilization, identifying predictors of diagnostic accuracy, and differentiating between remission and chronicity, as done in other fields such as oncology [15].

Given this premise, the aim of this paper is to describe (1) the challenges and pitfalls that were encountered in the process of adapting EHR data derived from the public mental health agency in Ferrara, Italy, for research purposes and (2) the development of a data set that is suitable for analysis via AI and traditional techniques. In order to test the feasibility of using these data in analyses and the robustness of analyses based on such data, a clustering analysis was also performed, and preliminary results are presented herein.

## Methods

### Ethics Approval

Ethical approval was obtained by the Area Vasta Emilia Centro Ethical Committee on December 12, 2019 (protocol number: 197/2018/Oss/AUSLF). This study conforms to the principles expressed in the Declaration of Helsinki.

### Setting

In Italy, mental health care is provided by departments of mental health [16-19]. The levels of care within each department of mental health include community-based mental health centers, hospital psychiatric inpatient units, and rehabilitation or residential facilities. Each community-based mental health center serves as a hub of psychiatric care for geographically defined catchment areas with 50,000 to 150,000 inhabitants [20] In Ferrara, Northern Italy, the Integrated Department of Mental Health and Pathological Addiction covers an area of 2630 km^²^, with a catchment of 342,061 inhabitants as of 2020 [21].

### Data Collection and EHRs

Data were collected in 2 periods that were distinct in terms of the methodology used, the psychiatric services delivered, and the level of digitalization. Data related to the first period, which began in 1925 and ended in 1990, were gathered mostly in a psychiatric asylum, during a time when digital health was not fully developed or adopted.

In 1991, the first structured EHR (ie, SIPER [Sistema Informativo Psichiatrico dell'Emilia-Romagna]) was introduced and implemented locally by the Local Health Trust of Ferrara for Mental Health in Adults. Different software programs were adopted during the years following the implementation of SIPER, and each new software program replaced the previous one by importing already existing data and adding new features (and thus information), as detailed in Textbox 1.

**Textbox 1. Electronic health records implemented by the Local Health Trust of Ferrara for Mental Health in Adults in chronological order.**

1. SIPER (Sistema Informativo Psichiatrico dell'Emilia-Romagna; 1991-1994) included individual demographic data, medical records, diagnoses, and health services.
2. CINECA (1994-1998) added the feature labeled as “project,” which was defined as the comprehensive set of treatments and activities offered to the patient.
3. GESAP (Gestione attività Psichiatrica; 1998-2004) added information about outpatient treatment; hospitalizations in inpatient units, long-term residences, and semiresidences; and outpatient services.
4. IPPOCRATE (GPI SRL; 2004-2008).
5. EFESO (Newteam SRL; 2008-2021) added the text field labeled “evaluation and treatment area” in the medical record, structured diagnostic evaluation, pharmacological treatment prescription and administration, clinical notes, attached documents, and a feature to identify structured clinical protocols (Percorso Diagnostico Terapeutico Assistenziale; diagnostic and therapeutic care pathway).
6. CURE (Cartella Unificata Regionale Elettronica; Engineering SpA; 2021 to present) added the registration of vital signs and laboratory tests, as well as legal and administrative documentation.

### Data Preparation

The first goal was the creation of a new, fully deidentified database with data available in EFESO (Newteam SRL)—a necessary step for complying with privacy constraints.

In order to remove all protected health information (PHI), source data needed to be modified. This could not be done by directly editing data in EFESO, since the source could not be altered directly. Thus, the new research database—the Ferrara-Psychiatry (FEPSY) database—was built via the Extract, Transform, Load process, which is a 3-phase process [22] in which data are first extracted from 1 source or multiple and possibly different sources (eg, databases, flat or formatted files, and web pages). Afterward, the extracted data are stored in a staging area, where they undergo transformation, such as filtering, cleaning, summarization, and normalization. Finally, the transformed data are loaded into the destination storage. For example, one type of transformation was record exclusion. We excluded records of patients that could not be unequivocally identified by the tax code—a unique 16-digit alphanumeric code that identifies a person in Italian public administration forms.

While assessing the suitability of the FEPSY database for research purposes, we noted that historical information dating back up to 1925 had also been maintained in EFESO. We understood that such data were manually imported into the electronic databases that preceded SIPER (year 1991); however, because we could not confirm the procedures, scope, and quality of this historical data import, we decided to document the existence of these data but exclude them from analysis.

More details about the FEPSY database can be found in Multimedia Appendix 1. In Table S1 in Multimedia Appendix 1, for each table in the FEPSY database, the total number of rows (number and percentage of records retained in the FEPSY database) is reported, alongside the number of records in the corresponding original EFESO table from which data were extracted (number of records in the corresponding EFESO table). As detailed in Table S1 in Multimedia Appendix 1, of the 4,264,954 records, 3,861,432 (90.54%) were kept. These records included detailed information about the patient, their illness, and the treatments provided.

In Table S2 in Multimedia Appendix 1, 2 types of anomalies for each table in the FEPSY database are described; one is date inconsistency (eg, when the closing date precedes the opening date of the medical chart), and the other is a date anomaly that was generated by the automatized mechanism that was introduced by EFESO when migrating data from IPPOCRATE (GPI SRL; August 26, 2008).

### Clustering

Once the anonymized database was built, a clustering analysis was performed to investigate the data set quality. A clustering algorithm is an unsupervised machine learning technique that is used to group objects, so that objects of the same group (or cluster) are very similar to one another and objects of different groups are very dissimilar. To decide the degree of similarity (or dissimilarity) between 2 objects, various distance measures can be used, such as the Euclidean distance between (normalized) numerical representations of the objects. We tested the hypothesis that the patients modeled in the data set could be divided into homogeneous clusters. The k-means algorithm computes a numerical distance between objects to determine to which cluster they belong. However, in our case, data were categorical. In 1995, Ralambondrainy [23] introduced an approach that enables the use of the k-means algorithm with categorical data. In this approach, nominal attributes are converted into binary attributes—one for each value that the attribute can take—so that they can be considered as numerical attributes by the algorithm.

We performed the clustering analysis with the WEKA (Waikato Environment for Knowledge Analysis; University of Waikato) data mining tool, which provides an implementation of the k-means algorithm (ie, SimpleKMeans) and can handle categorical data [24]. SimpleKMeans can also handle missing values by replacing them with the mean or mode.

For this preliminary clustering analysis, we included only the patients who had at least one recorded diagnosis of a mental disorder (ie, *International Classification of Diseases, Ninth Revision* [*ICD-9*] codes 290-319) [25].

Patients were excluded if they had nonpsychiatric diagnoses (*ICD-9* codes V01-V91; 2707/46,222, 5.86%) or had never received an *ICD-9* diagnosis (5493/46,222, 11.88%). The resulting subset for the clustering analysis included 38,022 patients.

We considered sociodemographic variables, such as biological sex, age at first visit, nationality, marital status, living situation, education, occupational status, birthplace (district), and the catchment area (district) providing care (determined by domicile postal code or by residence postal code when the domicile was missing).

## Results

### Sociodemographic Characteristics

The sample included 46,222 individuals, whose sociodemographic characteristics are detailed in Table 1.

**Table 1.** Sociodemographic characteristics of all of the individuals who accessed mental health services in the Ferrara province (1991-2021) and were included in the FEPSY^a^ database.

Characteristic		Female patients (n=28,109)	Male patients (n=18,113)	All patients (N=46,222)
**Age at first visit (years), mean (SD)**		50.46 (18.82)	48.72 (19.02)	49.78 (18.91)
	<18, n (%)	249 (0.89)	164 (0.91)	413 (0.89)
	18-24, n (%)	2173 (7.73)	1842 (10.17)	4015 (8.69)
	25-34, n (%)	4221 (15.02)	2913 (16.08)	7134 (15.43)
	35-44, n (%)	5141 (18.29)	3308 (18.26)	8449 (18.28)
	45-54, n (%)	4761 (16.94)	3119 (17.22)	7880 (17.05)
	55-64, n (%)	4142 (14.74)	2423 (13.38)	6565 (14.20)
	65-74, n (%)	3780 (13.45)	2180 (12.04)	5960 (12.89)
	≥75, n (%)	3634 (12.93)	2160 (11.93)	5794 (12.54)
	Missing data, n (%)	8 (0.03)	4 (0.02)	12 (0.03)
**Nationality, n (%)**				
	Italian	26,486 (94.23)	17,167 (94.78)	43,653 (94.44)
	Foreign	1580 (5.62)	920 (5.08)	2500 (5.41)
	Missing data	43 (0.15)	26 (0.14)	69 (0.15)
**Birthplace (district), n (%)**				
	Outside Ferrara province	7525 (26.77)	4825 (26.64)	12,350 (26.72)
	Ferrara	7299 (25.97)	5034 (27.79)	12,333 (26.68)
	Codigoro	3414 (12.15)	2167 (11.96)	5581 (12.07)
	Portomaggiore	2803 (9.97)	1795 (9.91)	4598 (9.95)
	Copparo	2439 (8.68)	1466 (8.09)	3905 (8.45)
	Cento	2262 (8.05)	1463 (8.08)	3725 (8.06)
	Outside Italy	2253 (8.02)	1258 (6.95)	3511 (7.60)
	Missing data	114 (0.41)	105 (0.58)	219 (0.47)
**Marital status, n (%)**				
	Married or partnered	10,748 (38.24)	6001 (33.13)	16,749 (36.24)
	Single	5551 (19.75)	5721 (31.59)	11,272 (24.39)
	Separated, divorced, or widowed	5587 (19.88)	1744 (9.63)	7331 (15.86)
	Missing data	6223 (22.14)	4647 (25.66)	10,870 (23.52)
**Living situation, n (%)**				
	Living with acquired family (partner and children)	12,147 (43.21)	6148 (33.94)	18,295 (39.58)
	Living with parents	3079 (10.95)	3338 (18.43)	6417 (13.88)
	Alone	3194 (11.36)	1768 (9.76)	4962 (10.74)
	Living with other family members	1649 (5.87)	781 (4.31)	2430 (5.26)
	Living with others (eg, roommates)	612 (2.18)	403 (2.22)	1015 (2.20)
	Community housing facilities	184 (0.65)	267 (1.47)	451 (0.98)
	Other	193 (0.69)	220 (1.21)	413 (0.89)
	Safe house	181 (0.64)	199 (1.10)	380 (0.82)
	Retirement home	226 (0.8)	146 (0.81)	372 (0.80)
	Prison	1 (3.56×10^−5^)	16 (0.09)	17 (0.04)
	Missing data	6643 (23.63)	4827 (26.65)	11,470 (24.82)
**Education, n (%)**				
	Illiterate	2941 (10.46)	1540 (8.50)	4481 (9.69)
	Literate (without formal degree)	3044 (10.83)	2312 (12.76)	5356 (11.59)
	Primary school	3674 (13.07)	2167 (11.96)	5841 (12.64)
	Middle school	3247 (11.55)	2644 (14.60)	5891 (12.75)
	High school	3946 (14.04)	2378 (13.13)	6324 (13.68)
	College or university	1294 (4.60)	584 (3.22)	1878 (4.06)
	Missing data	9963 (35.44)	6488 (35.82)	16,451 (35.59)
**Occupational status, n (%)**				
	Employed	3873 (13.78)	2735 (15.10)	6608 (14.30)
	Retired	2949 (10.49)	1833 (10.12)	4782 (10.35)
	Unemployed	1531 (5.45)	1291 (7.13)	2822 (6.11)
	Disability	612 (2.18)	670 (3.70)	1282 (2.77)
	Other	760 (2.70)	491 (2.71)	1251 (2.71)
	Homemaker	944 (3.36)	1 (0.01)	945 (2.04)
	Student	512 (1.82)	334 (1.84)	846 (1.83)
	Unknown	16,928 (60.22)	10,758 (59.39)	27,686 (59.90)
**Catchment area (district), n (%)**				
	Ferrara	10,964 (39.01)	6784 (37.45)	17,748 (38.40)
	Codigoro	4186 (14.89)	2931 (16.18)	7117 (15.40)
	Portomaggiore	3425 (12.18)	2193 (12.11)	5618 (12.15)
	Cento	3380 (12.02)	2097 (11.58)	5477 (11.85)
	Copparo	2754 (9.80)	1671 (9.23)	4425 (9.57)
	Unknown	3400 (12.10)	2437 (13.45)	5837 (12.63)

a FEPSY: Ferrara-Psychiatry.

### Extract, Transform, Load Process

#### Built-In Tables

Built-in tables from the EFESO relational database, which are detailed in Textbox 2, were included in the FEPSY database.

**Textbox 2. Built-in tables included in the FEPSY (Ferrara-Psychiatry) database.**

1. Table *Patients* included individual personal data, such as name, place and date of birth, biological sex (male or female), home address, living condition, education, marital status, occupation, and other sociodemographic characteristics.
2. Table *Medical Records* contained 1 or more medical records for each patient, with information such as the date of admission, date and type of discharge, primary diagnosis of a mental disorder, and facility providing care.
3. Table *Diagnoses* included 1 or more diagnoses that were assigned to each individual. Diagnoses were classified according to the *International Classification of Diseases, Ninth Revision* (*ICD-9*) categorical system; therefore, every diagnosis included the associated *ICD-9* code, description, group, and chapter [24]. Diagnoses recorded before the introduction of the *ICD-9* were recorded in SIPER (Sistema Informativo Psichiatrico dell'Emilia-Romagna), using standardized conversion criteria [24].
4. Table *Products* referred to the different types of medical services, such as consultations or hospitalizations. A product had a start date and end date, and it may have contained 1 or more medical services.
5. Table *Medical Services* stored every service that each individual had received or undergone, such as consultations, first visits, the administration of pharmacological treatment, social skill–oriented activities, structured diagnostic assessments, and mandatory medical treatments, as well as the facility providing care.
6. Tables *Medication Prescription* and *Medication Administration* referred to the prescription and administration of pharmacological treatment, type of medication and dosage, start and stop dates, and responsible facility.
7. Table *Psychometric Tests* included every test administered to each patient and the test types, dates, questions, and scores.
8. Table *Projects* listed the treatment plans for each patient. There were individual and group projects, and within a project, there could have been 1 or more products and medical services.
9. Table *Facilities* contained all of the facilities of the Health Trust of Ferrara for Mental Health in Adults, such as hospitals, day care centers, and clinics, along with their types and locations.

#### Extract

Data were extracted from EFESO by using an automated procedure that executed an SQL select query. This query selected all relevant fields of a table and other useful information from linked support tables, such as the descriptions of the codes. The result of the query was stored in a Pandas DataFrame (The Pandas Development Team) [26,27], which can be easily manipulated in the next phase.

#### Transform

##### Record Exclusion

Data imported before 1991 were excluded (as detailed in the *Data Preparation* section). Some fields and records were removed [22] to ensure data consistency, because there were duplicate or erroneous records (Table S1 in Multimedia Appendix 1). These were (1) fields containing unreliable information, (2) fields that were present but not in use (their values were always null), and (3) all records marked as “deleted” (ie, wrong records that were not to be used) and all records in other tables referencing the “deleted” ones. We decided to remove 36 individuals that had unique fiscal codes but duplicate patient IDs—corresponding to 0.15% (72/48,001) of the records in the total data set—since it was not possible to determine which of the two entries was the correct one. When a patient was first included in the database, a unique identifier—the patient ID—was assigned. The combination of the tax code and the patient ID allowed for the unique identification of a patient in the database. We also excluded patients for whom a record was opened earlier than the birth date (16/48,001, 0.03%), patients with no medical records (603/48,001, 1.26%), and patients marked as “deleted” (77/48,001, 0.16%). Overall, 1.6% (768/48,001) of the total records, which related to 732 patients, were removed from the source table.

##### Anonymization

Anonymization was necessary in order to use the extracted data for research projects and was performed on tables *Patients* and *Medical Records*. First, the extracted records were shuffled. Afterward, the original patient and medical record IDs were replaced with a universally unique identifier (UUID), which is a 128-bit string that is usually represented as a sequence of 32 hexadecimal digits [28]. These new random, unique identifiers were generated with the *uuid4* function of the Python *uuid* package (Python Software Foundation) [29] and used as the new primary key. In order to maintain the referential integrity (ie, the primary key of one table is a foreign key in another table, meaning that they are related), the old IDs were replaced with the new ones within every table in which they appeared. Furthermore, all PHI were excluded from table *Patients*; these data included first names, last names, days and months of birth, tax codes, home addresses, phone numbers, and note fields that could potentially include personal data (eg, relatives’ names). For the same reason, text note fields were also excluded from other tables, when present.

##### Field Transformation

Transformation was necessary for date fields. EFESO stored dates in “datetime” format, that is, “dd/mm/yyyy hh:mm.” However, previous EHRs stored only the date, without the hour information. Furthermore, even when specified, the hour information is not always reliable. For this reason, the date fields were split into 2 fields—one for the date and one for the time.

##### Missing Values

Missing values were assessed to avoid the introduction of bias. In specific analyses, the level and pattern of missingness will be assessed for each variable included and dealt with accordingly.

### Load

Data extracted from EFESO were loaded in the FEPSY database—the newly created MySQL (Oracle Corporation) relational database—by using the same automated procedure that was used to extract them. For each built-in table, an insert query, which took the values from the same DataFrame of the select query, was executed.

### Analysis of the Extracted Data

**Figure 1.**
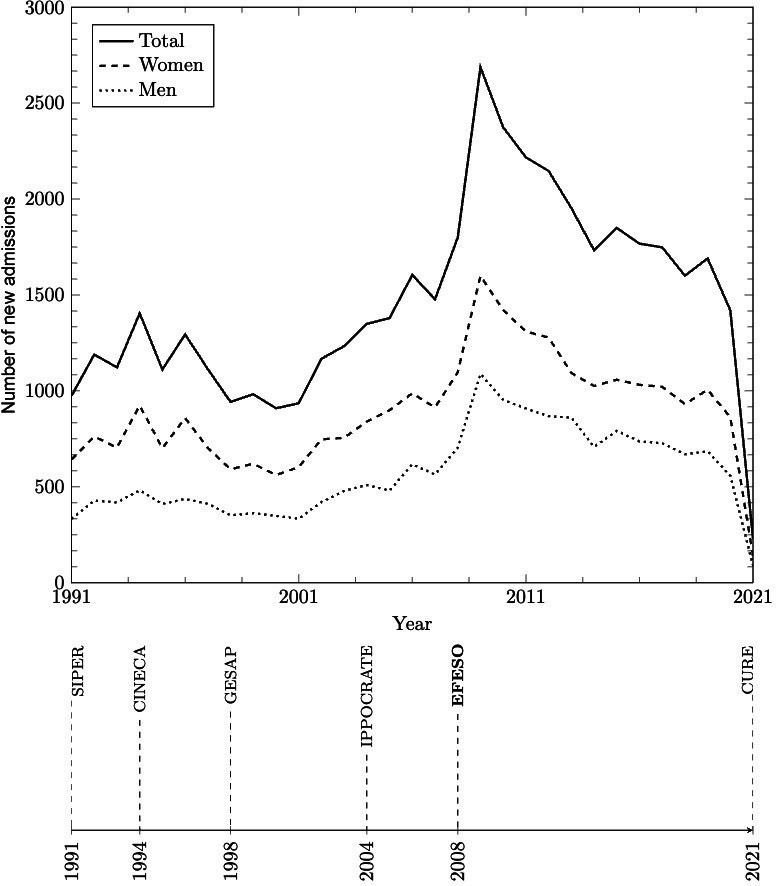
New admissions per year in total and by sex (upper panel). Timeline of the electronic health records adopted by the health care agency in the Ferrara province (lower panel). CURE: Cartella Unificata Regionale Elettronica; GESAP: Gestione attività Psichiatrica; SIPER: Sistema Informativo Psichiatrico dell'Emilia-Romagna.

**Figure 2.**
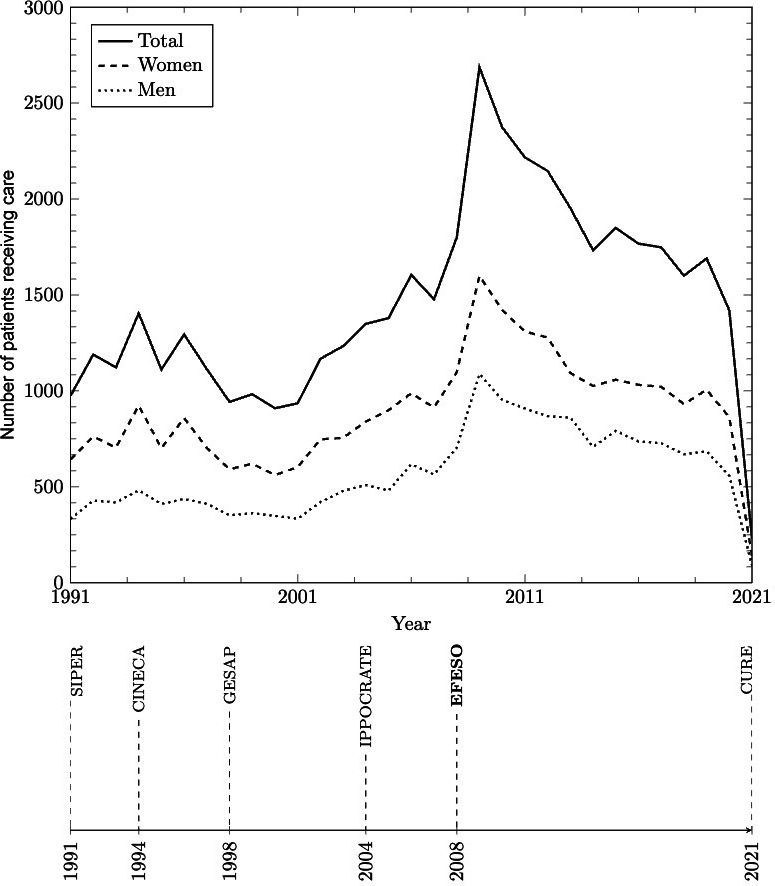
Patients receiving care from the mental health services in Ferrara over time (years 1991-2021), in total (continuous line) and by sex (dotted and dashed lines). CURE: Cartella Unificata Regionale Elettronica; GESAP: Gestione attività Psichiatrica. SIPER: Sistema Informativo Psichiatrico dell'Emilia-Romagna.

The data included in the final composite FEPSY data set were those collected from 1991 to February 2021. Since 1991, each year, a median of 1404 (IQR 1117.5-1757.7) individuals had newly accessed care, and a median of 7300 (IQR 6109.5-9397.5) individuals were actively receiving care, as represented in Figure 1. Figure 2 shows the number of patients treated per year in total and by sex. The sudden decrease observed in 2009 was due to an automated closing procedure that was introduced in 2008 and retained from then on. When migrating from IPPOCRATE to EFESO, all medical records, products, and diagnoses that had not been updated in 365 days were assumed to be closed or terminated, and the missing closing date was replaced with the date of the migration to EFESO, that is, August 26, 2008.

As described in Table 2, the most frequent diagnoses at first admission were depression and anxiety disorder. During the 30-year time span, more than half (32,230/46,222, 69.73%) of the patients had only 1 chart open, and only 5184 patients had at least one psychiatric hospitalization.

**Table 2.** Main clinical characteristics of the sample (N=46,222; years 1991-2021).

Characteristics		Female patients (n=28,109, 68.81%)	Male patients (n=18,113, 39.19%)
**Age at first visit**			
	Years, mean (SD)	50.46 (18.82)	48.72 (19.02)
	Years, median (range)	49.0 (0-109)	47.0 (2-98)
**Number of charts/patient**			
	Value, mean (SD)	1.62 (1.55)	1.64 (2.38)
	Value, median (range)	1.0 (1-63)	1.0 (1-132)
Patients with at least one hospitalization, n		2680	2504
**Number of hospitalizations/patient**			
	Value, mean (SD)	0.33 (2.36)	0.46 (2.35)
	Value, median (range)	0 (0-143)	0 (0-102)
Patients with at least one compulsory admission, n (%)		415 (1.48)	485 (2.68)
**Duration of hospitalization**			
	Days, mean (SD)	5.08 (40.89)	7.53 (62.39)
	Days, median (range)	0 (0-2661)	0 (0-4090)
**First recorded mental disorder diagnosis^a^,** **n (%)**			
	Anxiety disorders	6884 (24.49)	3725 (20.57)
	Dementia and other organic disorders	2092 (7.44)	1601 (8.84)
	Depression	7648 (27.21)	3335 (18.41)
	Drug and substance use or abuse	415 (1.48)	861 (4.75)
	Eating disorders	241 (0.86)	18 (0.10)
	Intellectual disability	468 (1.66)	636 (3.51)
	Mania and bipolar disorders	713 (2.54)	460 (2.54)
	Personality disorders	1287 (4.58)	1186 (6.55)
	Schizophrenia and other nonorganic psychoses	1468 (5.22)	1515 (8.36)
	Other mental disorders	2339 (8.32)	1130 (6.24)
	No formal mental disorder diagnosis	4554 (16.20)	3646 (20.13)

a Mental disorder diagnoses: *International Classification of Diseases, Ninth Revision* codes 290.xx-319.xx.

### Clustering Results

**Figure 3.**
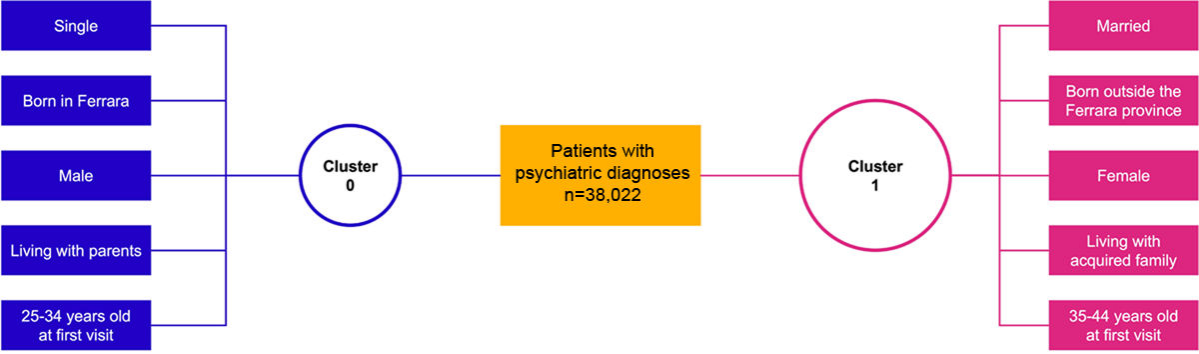
Results from the clustering analysis.

This analysis, which was carried out on the subset of 38,022 individuals who had at least one mental disorder diagnosis, identified 2 distinct clusters (Figure 3). One is represented by single male patients who were born in Ferrara, those who were living with parents, and those whose age at first visit was between 25 and 34 years; the other is represented by married female patients who were living with their own acquired families, those who were born outside the province of Ferrara, and those whose age at first visit was 35 to 44 years. The following sociodemographic features were similar in the two clusters: Italian nationality, individuals with a high school degree, employed individuals, and individuals who were receiving treatment in the Ferrara catchment area.

## Discussion

### Principal Findings

This study describes the process of adapting one of the longest running EHRs of public mental health care for research purposes. The FEPSY data set covers a catchment area with 342,061 inhabitants (as of 2020) and includes a total of 46,222 unique individuals who had access to mental health services over a span of 30 years (1991-2021). The FEPSY database is suitable for descriptive, predictive, and inferential analyses via conventional analysis and AI techniques, as demonstrated by the preliminary findings of the clustering analysis. To our knowledge, our database is the first of its kind in Italy. In Europe, longitudinal and prospective registries have long been in use. For example, large data sets were extracted from the Danish National Patient Registry [30] and the Danish Psychiatric Central Research Register [31]. The first data set contained data on 8,085,603 patients, which were collected from 1977 to 2012. The second data set included data on a total of 747,176 patients, which were collected from 1970 to 2010. In both cases, the register contained dates of the onset and end of any treatment, diagnoses, types of referrals, and places of treatment, thereby allowing for the possibility to perform health registry–based research [32], considering a total population of approximately 27 million.

The main finding of this study is that data that were not originally conceived for research were successfully extracted from EHR software and loaded into a new anonymized database. This step is of foremost importance, as the data originated from an information system that was changed and updated multiple times and was not designed to allow for exploratory investigations in a structured manner. Thus, this new data set may represent the ideal setting to build, test, and refine an analytical methodology for extracting data and preparing these data for research purposes. This methodology could also be applied to other clinical data sets, such as data sets from other medical disciplines (eg, oncology), with characteristics that are similar to those of the FEPSY data set [33]. Additionally, it will be of foremost importance to validate the methodological approach and findings of upcoming research originating from the FEPSY data set by proactively seeking collaboration with other research groups, in order to enable the replication of findings from the territory of Ferrara and the use of the FEPSY data set to replicate findings from research involving other registries.

This work also allows for collaborations in terms of learning health networks, which use comparable data that originate from EHRs to support clinical decisions, improve the delivery of efficient and effective medical care, and help with the integration of research in health care [34-36].

Our results will also pave the way for an in-depth study on the use of health care resources; the results will be used to develop a system that is capable of planning the use of such resources. Such a system would optimize the use of health care resources while maintaining or possibly improving the quality of treatment. For example, in Italy, Donisi et al [37] predicted the cost of community mental health care by using clinical and sociodemographic information originating from the Psychiatric Case Register in the Verona Health District. This allowed for the linking of social deprivation to psychiatric service utilization [38] and shed a light on possible contributors to social isolation in an already vulnerable population. Our clustering analysis, which was conducted on the FEPSY data set to test its feasibility and robustness, identified 2 clusters; women appeared to access mental health services later in life and were typically married, in comparison to men. These findings were consistent with the literature [39-41] and supported the ability of the extracted data to detect known patterns, even though the results should be interpreted with caution, given the large amount of missing sociodemographic data. Clustering analyses can be useful for building prediction models and planning a department's resource allocation, as they provide relevant information on patients at presentation and on illness trajectory [42].

The process described in this paper faced 5 major challenges that we mitigated, as follows. First, the source data included several built-in structural informatic elements (so-called *tables*) that had to be screened and deleted in order to get to the core data. Second, the anonymization step was of absolute importance, and in order to both comply with privacy constraints and be able to preserve the integrity of the data, the study team decided to keep only subelements of certain data items (eg, for the birth date, only the year was kept, and for residence, only the postal code was kept). Third, in order to establish which records were correct, an iterative comparison of the FEPSY database and the local and regional database was performed by a third party who had access to PHI. Fourth, records that were deemed unreliable were excluded (eg, clinical procedures referring to nonexistent medical records). Furthermore, the data extracted from EFESO originated from different software and thus possibly generated some errors. In the FEPSY data set, these errors seem to be limited to records, and the proportion of records with errors was very low (49,854/3,861,432, 1.29%). In the end, we decided to exclude data that were collected before 1991 and duplicate patient records. Fifth, missing data challenges were also addressed, especially in the clustering process. For this purpose, the WEKA data mining tool was used.

### Strengths of This Study

The quality and completeness of the collected and cleaned data, as well as the large number of records stored in the FEPSY database, resulted in the definition of a data set that is particularly suitable for automatic analysis and has appealing characteristics for research, such as a long period of data availability, great diversity in the sociodemographic factors of the patients represented, and a history of treatments and drugs administered. This could possibly represent a strong foundation for many different studies of mental illness and resource use, favoring comparisons between Italy and other countries regarding the delivery and quality of community and hospital psychiatric care [43-45]. Furthermore, the newly created database does not include sensitive information, even though this information can be retrieved by using an external supporting table created ad hoc, which could link the FEPSY data set with other data sets (eg, hospital data and tumor registries) for future research.

The novelty of this project is represented by its interdisciplinary nature (psychiatry, public health, epidemiology, sociology, mathematics, computer science, and AI), the potential versatility of the methods that can be used with the FEPSY database, and the versatility of the systems that could be created via analyses involving the FEPSY database. To our knowledge, this study is the first attempt to retrospectively build a single data set that includes more than 30 years’ worth of data on mental health services in a specific area.

Such a data set would also allow for longitudinal analyses, such as those that have already been performed with the Nordic registry (a prospective registry) and, more recently, the South London and Maudsley National Health Service [46] and the Camden & Islington Research Database [47].

We believe that historical data can add value to subsequent analyses, because they allow researchers to understand how mental health services have evolved over the past decades and the extent to which phenotypical presentations of different diseases have changed over time. In light of these considerations, factors that should be taken into account are (1) potential cohort and time effects, such as historical events (eg, the Great Recession in 2008); (2) changes in legal and medical approaches to mental health; and (3) changes in the classification of mental disorders [48].

### Limitations

Our findings must be interpreted in the light of some limitations. First, the sample size is limited by the geographical catchment. A larger catchment or a more densely populated region would probably have a larger volume of treated individuals and thus have more data, which would facilitate machine learning analyses. However, we believe that even if the sample is limited by the geographical catchment, the diverse socioeconomic distribution in Ferrara is a strength that mitigates this limitation, providing insight into the possible moderator or mediator roles of socioeconomic variables that are considered social determinants of mental health. Second, another potential limitation is the missing data for some sociodemographic attributes, which may reduce the statistical power of a study [49] or affect the accuracy of machine learning algorithms [50]. In order to overcome this issue, missing values can be handled with multiple imputation methods or replaced with the mean or the mode (ie, for quantitative or qualitative data, respectively). Moreover, sociodemographic information can be drawn from external and publicly available sources, such as the Italian National Institute of Statistics [51], which includes the census of the population as well as social, economic, and environmental surveys and analyses. Lastly, there is the risk of introducing bias while building prediction models, especially when using supervised machine learning techniques, due to small sample sizes and the poor handling of missing data and overfitting [52]. With regard to the sample size, our sample appears to be sufficiently large for risk prediction analyses. Overfitting can be addressed with penalized models [53].

### Future Directions

This work sets a starting point for future investigations, which can be described as follows: (1) identifying patients who have a higher severity index or chronicity level and those who require a greater use of health resources; (2) identifying and validating, by means of machine learning models, demographic, clinical, and social predictors of clinically relevant outcomes that are useful for an ad hoc programming of resources (eg, sex, gender, or social deprivation [38,39,54]); (3) further optimizing and tailoring the analysis methods, so that they can also be applied to other data sets (eg, the local mental health registries for child and adolescent neuropsychiatry and for drug addiction services); (4) interacting with international learning health networks [55-57]; and (5) linking the FEPSY data set with external data sources, such as census data, tumor registries [58], death registries, and criminal justice data [59]. As a result of the increasing digitalization of medical records, it was possible to gather years of mental health history for every patient. This will enable for the conduct of symbolic and subsymbolic analyses on time series via automatic methodologies. Classical supervised and unsupervised machine learning and deep learning techniques will be evaluated. In order to explore the relationship between sociodemographic characteristics and specific diagnostic questions (eg, the incidence and prevalence of psychosis), a supervised framework will be deployed, in which binary labels (eg, “psychosis” or “no-psychosis”) or multiple classification labels (eg, “ICD-9 diagnosis”) will be associated with the patient. The main problem to overcome will be the imbalance of the data set, that is, when there is an unequal distribution of classes in the data set. In such instances, a standard machine learning technique, such as a support vector machine or random forest [60,61], will be applied. Moreover, each patient could potentially be considered as a distinct time series by including the temporal dimension of the treatment and by applying recurrent neural networks [62,63]. By doing so, the prediction of the new onset of a disease and the subsequent use of health resources will be the focus, in order to plan and optimize health care resources.

### Conclusions

The process described in this study resulted in the building of a data set that included the information of 46,222 individuals who had access to psychiatric services in the Ferrara province over the course of almost 30 years. The preliminary findings from the clustering analysis confirmed the quality of the newly established database. The process we implemented proved to be a solid method that can be replicated with similar data sets, even if they were not originally compiled for research purposes.

## Supplementary material

10.2196/45523 Multimedia Appendix 1 Characteristics of the records within the tables of the Ferrara-Psychiatry (FEPSY) database.

## Abbreviations

AI: artificial intelligence
EHR: electronic health record
FEPSY: Ferrara-Psychiatry
*ICD-9*: *International Classification of Diseases, Ninth Revision*
PHI: protected health information
SIPER: Sistema Informativo Psichiatrico dell'Emilia-Romagna
UUID: universally unique identifier
WEKA: Waikato Environment for Knowledge Analysis
