# Supplementary material for: Establishment of a Public Mental Health Database for Research Purposes in the Ferrara Province: Development and Preliminary Evaluation Study
Source: JMIR Med Inform. 2023 Aug 9;11:e45523. doi: 10.2196/45523 (PMC10461404; doi:10.2196/45523)
Supplement: Multimedia Appendix 1 [file medinform-v11-e45523-s001.docx]

**Multimedia Appendix**

**Multimedia Appendix Table S1.** Extraction results: for each Table of the FEPSY database the number of retained records and the number of records of the original table in EFESO are reported. Data range from 1991 to February 2021.

| Table in FEPSY database | Records in the corresponding EFESO's table | Records retained in the FEPSY database |
| --- | --- | --- |
|  | N | n(%) |
|  |  |  |
| **Diagnoses** | 128720 | 121349 (94.27) |
|  |  |  |
| **Facilities** | 237 | 237 (100.0) |
|  |  |  |
| **Medical Records** | 79820 | 75173 (94.18) |
|  |  |  |
| **Medical Services** | 2910586 | 2584797 (88.81) |
|  |  |  |
| **Medication Administrations** | 668259 | 633088 (94.74) |
|  |  |  |
| **Medication Prescriptions** | 167353 | 159318 (95.20) |
|  |  |  |
| **Patients** | 48001 | 46222 (96.29) |
|  |  |  |
| **Products** | 207879 | 191511 (92.13) |
|  |  |  |
| **Projects (Treatment plans)** | 49009 | 44713 (91.23) |
|  |  |  |
| **Psychometric Tests** | 5090 | 5024 (98.70) |
|  |  |  |

**Multimedia Appendix Table S2.** Report on data anomalies on the original dataset: this includes dates inconsistency (i.e., wrong order of opening/start and closing/end dates), records in which a default date (i.e., the date when the migration to EFESO occurred) was inserted to replace missing dates, records with missing date information. Data range from 1991 to February 2021.

|  | Number of records with | | Errors on 3,861,432 records |
| --- | --- | --- | --- |
| Category Table | Dates inconsistency | EFESO migration default date | n(%) |
|  |  |  |  |
| **Diagnoses** | 6691 | 19385 | 25546 (0.66) |
|  |  |  |  |
| **Medical Records** | 784 | 11070 | 11854 (0.31) |
|  |  |  |  |
| **Medical Services** | - | 224 | 224 (0.01) |
|  |  |  |  |
| **Medication Administration** | - | 0 | 0 (0.00) |
|  |  |  |  |
| **Medication Prescription** | - | 0 | 0 (0.00) |
|  |  |  |  |
| **Medication Prescription** (start and stop dates) | 915 | 0 | 915 (0.02) |
|  |  |  |  |
| **Patients** | - | - | 0 (0.00) |
|  |  |  |  |
| **Products** | 37 | 27 | 64 (0.00) |
|  |  |  |  |
| **Projects**  **(Treatment plans)** | 64 | 11038 | 11251 (0.29) |
|  |  |  |  |
| **Psychometric Tests** | - | 0 | 0 (0.00) |
|  |  |  |  |
